# Supplementary material for: Inhibition of the proton-activated chloride channel PAC by PIP2
Source: eLife. 2023 Jan 12;12:e83935. doi: 10.7554/eLife.83935 (PMC9876566; doi:10.7554/eLife.83935)
Supplement: Supplementary file 1. [file elife-83935-supp1.docx]

**Supplementary file 1: Cryo-EM data collection, refinement, and validation statistics**

| ‍ | **Human PAC with diC8-PI(4,5)P_2_ at pH 4.0** | |
| --- | --- | --- |
|  | **Full PAC map** | **Focused map on PAC ECD and the extracellular region of TMD** |
| **Data collection and processing** |  |  |
| Magnification | 105,000 | 105,000 |
| Voltage (kV) | 300 | 300 |
| Electron exposure (e^–^/Å^2^) | 50 | 50 |
| Defocus range (μm) | –0.6 to –2.4 | –0.6 to –2.4 |
| Pixel size (Å) | 0.826 | 0.826 |
| Symmetry imposed | C3 | C3 |
| Initial particle images (no.) | 5,859,347 | 5,859,347 |
| Final particle images (no.) | 84,149 | 84,149 |
| Map resolution (Å)  FSC threshold | 2.71  0.143 | 2.70  0.143 |
| Map resolution range (Å) | 2.2 – 8.9 | 2.2 – 6.9 |
|  |  |  |
| **Refinement** |  |  |
| Initial model used (PDB code) | *7SQH* | *7SQH* |
| Model resolution (Å)  FSC threshold | 3.04  0.5 | 3.05  0.5 |
| Map sharpening *B* factor (Å^2^) | –95 | –96.3 |
| Model composition  Non-hydrogen atoms  Protein residues  Ligands | 6903  849  15 | 6822  834  15 |
| R.m.s. deviations  Bond lengths (Å)  Bond angles (°) | 0.003  0.518 | 0.003  0.521 |
| Validation  MolProbity score  Clashscore  Poor rotamers (%) | 1.40  7.24  0.00 | 1.40  7.23  0.00 |
| Ramachandran plot  Favored (%)  Allowed (%)  Disallowed (%) | 98.93  1.07  0.00 | 98.91  1.09  0.00 |
